# Supplementary material for: Inflammatory disease progression shapes nanoparticle biomolecular corona-mediated immune activation profiles
Source: Nat Commun. 2025 Jan 22;16:924. doi: 10.1038/s41467-025-56210-4 (PMC11754911; doi:10.1038/s41467-025-56210-4)
Supplement: Supplementary file 1 — Supplementary Information [file 41467_2025_56210_MOESM1_ESM.pdf]

**Supplementary Information**

# Inflammatory Disease Progression Shapes Nanoparticle Biomolecular Corona-Mediated Immune Activation Profiles

Jacob R. Shaw[1], Nicholas Caprio[2], Nhu Truong[2], Mehari Weldemariam[2], Anh Tran[2], Nageswara Pilli[2], Swarnima Pandey[2], Jace W. Jones[2], Maureen A. Kane[2,3], Ryan M. Pearson[1,2,3,*]

1. Department of Microbiology and Immunology, University of Maryland School of Medicine, 685 W. Baltimore Street, Baltimore, MD 21201, US
2. Department of Pharmaceutical Sciences, University of Maryland School of Pharmacy, 20 N. Pine Street, Baltimore, MD 21201, US
3. Marlene and Stewart Greenebaum Comprehensive Cancer Center, University of Maryland School of Medicine, 22 S. Greene Street, Baltimore, MD 21201, US

[*]Address correspondence to:
Ryan M. Pearson, Ph.D.
Associate Professor, Department of Pharmaceutical Sciences
Director, Bio- and Nano-Technology Center
University of Maryland School of Pharmacy
20 N. Pine Street
N525 Pharmacy Hall
Baltimore, MD 21201, USA
Phone: 410-706-3257

Email: rpearson@rx.umaryland.edu

KEYWORDS: Inflammation, biomolecular corona, immunomodulation, nanoparticles, immune activation

| Formulation | Corona Coating | Size Mean (nm) | Size Mode (nm) | PDI | Zeta Potenial (mV) |
|---|---|---|---|---|---|
| PLGA-PEMA | n/a | 165.2 ± 65.8 | 157.9 | 0.102 ± 0.023 | -55 ± 1.2 |
| PLGA-PEMA | Naïve Plasma | 246.1 ± 62.3 | 205.6 | 0.355 ± 0.033 | -27.2 ± 1.5 |
| PLGA-PEMA | 3hr LPS Plasma | 238.1 ± 81.3 | 201.7 | 0.357 ± 0.018 | -28.0 ± 0.8 |
| PLGA-PEMA | 8hr LPS Plasma | 258.2 ± 86.9 | 170.4 | 0.378 ± 0.070 | -23.9 ± 1.2 |
| PLGA:PEG5k-PEMA | n/a | 110.3 ± 75.5 | 121.8 | 0.155 ± 0.009 | -34 ± 0.6 |
| PLGA:PEG5k-PEMA | Naïve Plasma | 166.3 ± 64.7 | 119.8 | 0.398 ± 0.021 | -28.7 ± 1.2 |
| PLGA:PEG5k-PEMA | 3hr LPS Plasma | 163.6 ± 68.9 | 115.7 | 0.443 ± 0.010 | -36.1 ± 5.7 |
| PLGA:PEG5k-PEMA | 8hr LPS Plasma | 143.8 ± 67.1 | 106.8 | 0.424 ± 0.013 | -32.0 ± 0.8 |
| PLA-PEMA | n/a | 209.8 ± 59.2 | 186.4 | 0.118 ± 0.039 | -34 ± 1.4 |
| PLA-PEMA | Naïve Plasma | 299.8 ± 86.7 | 219.1 | 0.730 ± 0.110 | -24.4 ± 0.5 |
| PLA-PEMA | 3hr LPS Plasma | 238.6 ± 77.7 | 150.3 | 0.654 ± 0.134 | -25.0 ± 1.7 |
| PLA-PEMA | 8hr LPS Plasma | 266.2 ± 74.8 | 216.4 | 0.437 ± 0.072 | -25.7 ± 1.4 |
| PLA-PVA | n/a | 175.5 ± 1.8 | 142.7 | 0.099 ± 0.006 | -6.37 ± 0.07 |

**Supplementary Table 1: Nanoparticle corona physicochemical properties.**

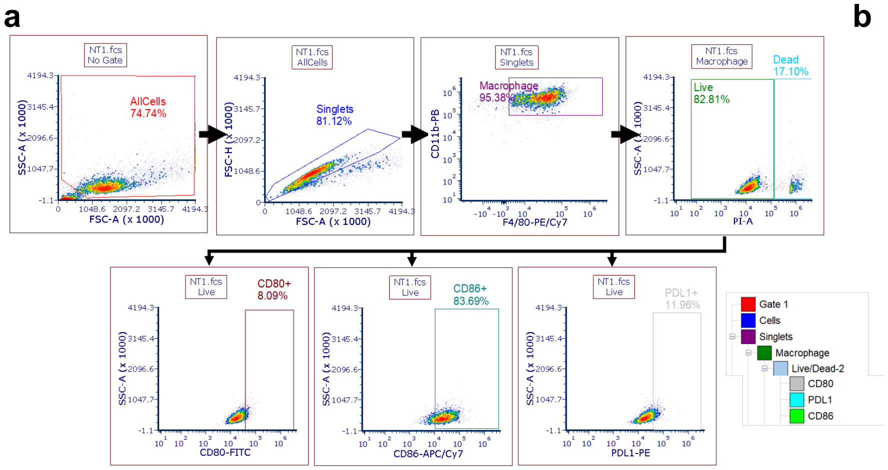


**Supplementary Fig. 1: Flow cytometry of NP corona-stimulated macrophages**. **a.** Representative flow cytometry gating strategy of No Treatment (NT) macrophages for receptor identification. **b.** Percent of live macrophages after treatment with PLGA NPs or NP coronas for 24 hours. Live/Dead was determined with PI staining. Significance was determined using a one-way ANOVA with Tukey's post-hoc test. Data sets are presented as mean ± S.D of n = 3 biological replicates with 10,000 events each). *P < 0.05; **P < 0.01; ***P < 0.001, ****P < 0.0001. ns, not significant (P > 0.05). Source data are provided as a Source Data file.

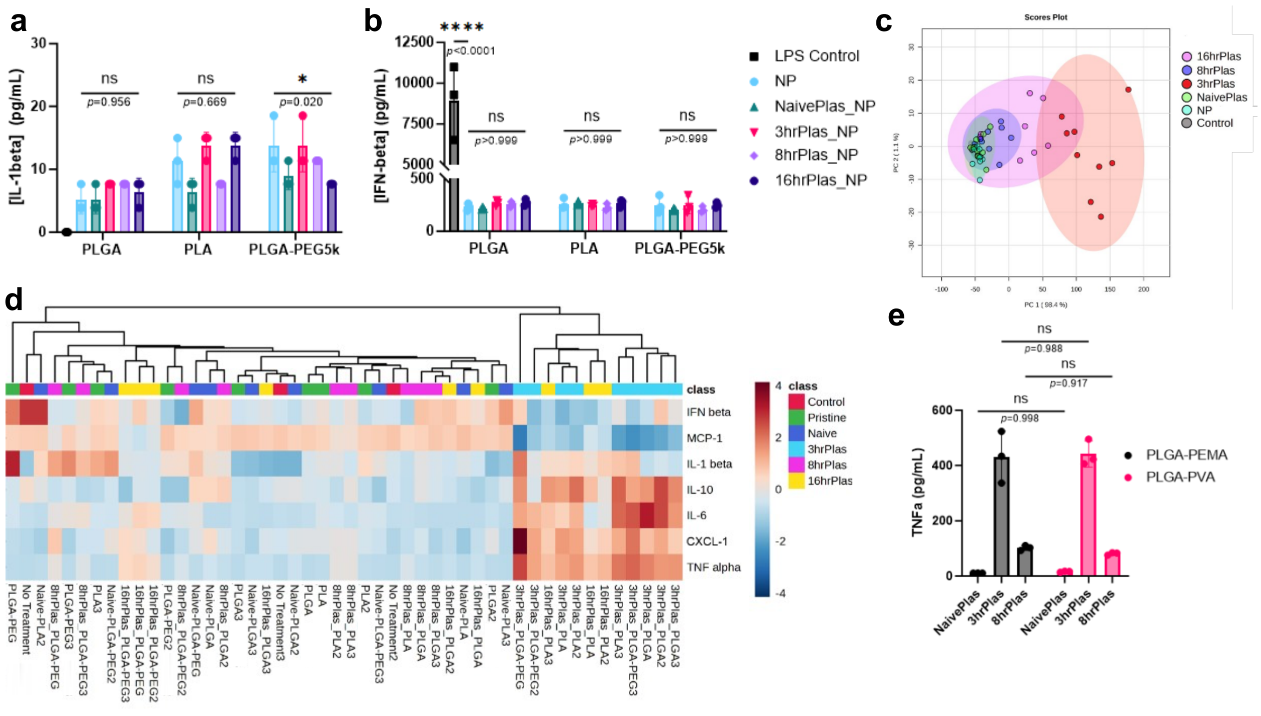


**Supplementary Fig. 2: NP corona-induced macrophage cytokine secretions**. Multiplex cytokine analysis in the supernatants of NP corona treated bone marrow-derived macrophages after 3-hour incubation. IL-1β (**a**) and IFN-β (**b**) are represented with statistical significance determined by a two-way ANOVA using Tukey's post-hoc test. **c.** Principal component analysis of corona-induced cytokine responses from the three core polymers and 5 different plasma coatings (n = 3 per condition). Non-treated macrophage are labeled as "Control". **d**. Heatmap representation of the multiplex cytokine analysis. **e.** Evaluation of NP surfactant influence on corona-dependent macrophage TNFα expression using poly(vinyl alcohol) (PVA). Significance was determined using a two-way ANOVA with Šídák post-hoc test. Data sets are presented as mean ± S.D of n = 3 biological replicates with 10,000 events each. *P < 0.05; **P < 0.01; ***P < 0.001, ****P < 0.0001. ns, not significant (P > 0.05). Source data are provided as a Source Data file.

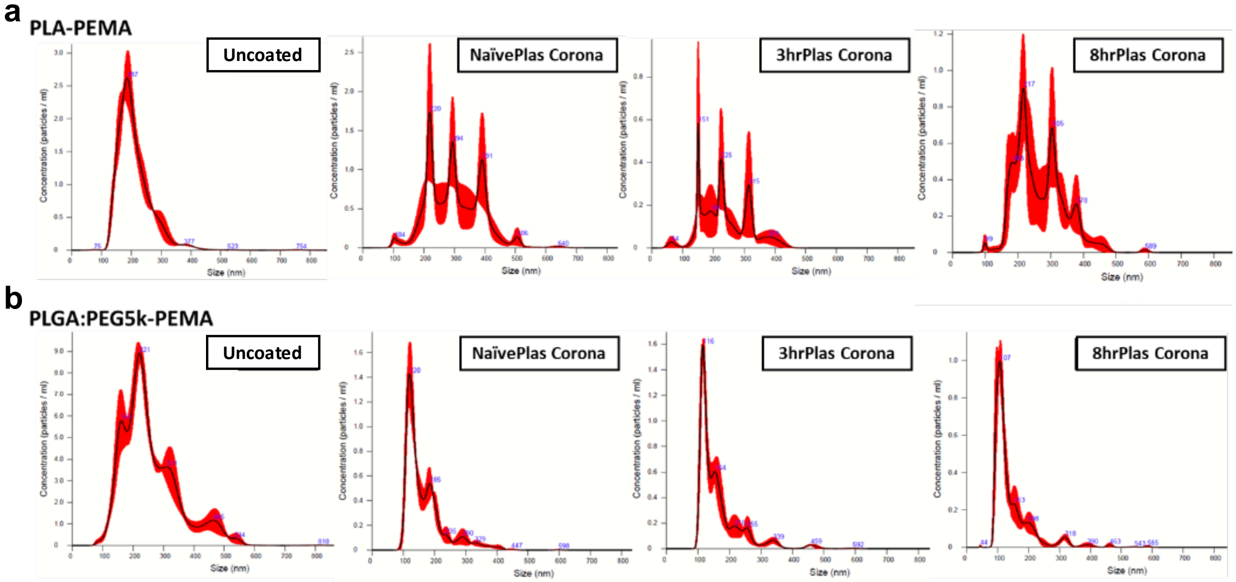


**Supplementary Fig. 3: Polymer-dependent NP corona size characterization**. Nanoparticle tracking analysis quantification of PLA (**a**) or PLGA-PEG5k (**b**) nanoparticle size distributions with varying coronas post-washing. Plots are representative of n = 3 measurements of 30 second recordings. Error bars are represented as red shaded regions.

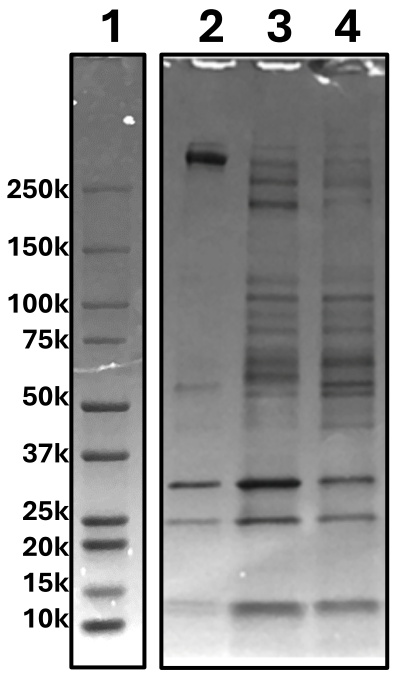


**Supplementary Fig. 4: PEGylated PLGA corona protein quantification SDS-PAGE**. Coomassie stained SDS-PAGE gel of PLGA NP corona proteins compared to PEGylated PLGA. Lanes: (1) Protein Ladder; (2) PLGA-PEG5k_NaïvePlas; (3) PLGA_NaïvePlas; (4) PLGA_3 hrPlas. Source data are provided as a Source Data file.

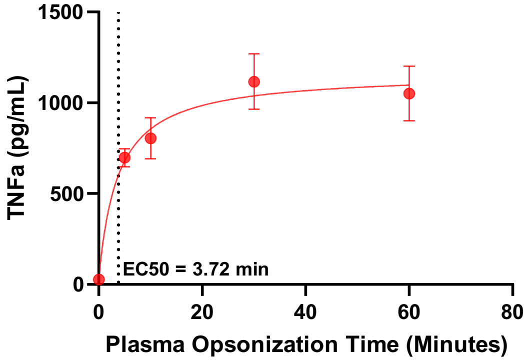


**Supplementary Fig. 5: Pro-inflammatory coronas form rapidly upon NP introduction**. TNFα secretions from macrophages after incubation for 3-hours with PLGA NPs that were opsonized in 3hr-post LPS plasmas for varying times. EC50 is shown to illustrate half-maximal TNFα induction after 3.72 minutes opsonization of NPs in plasma. Data sets are presented as mean ± S.D of n = 3 biological replicates. Source data are provided as a Source Data file.

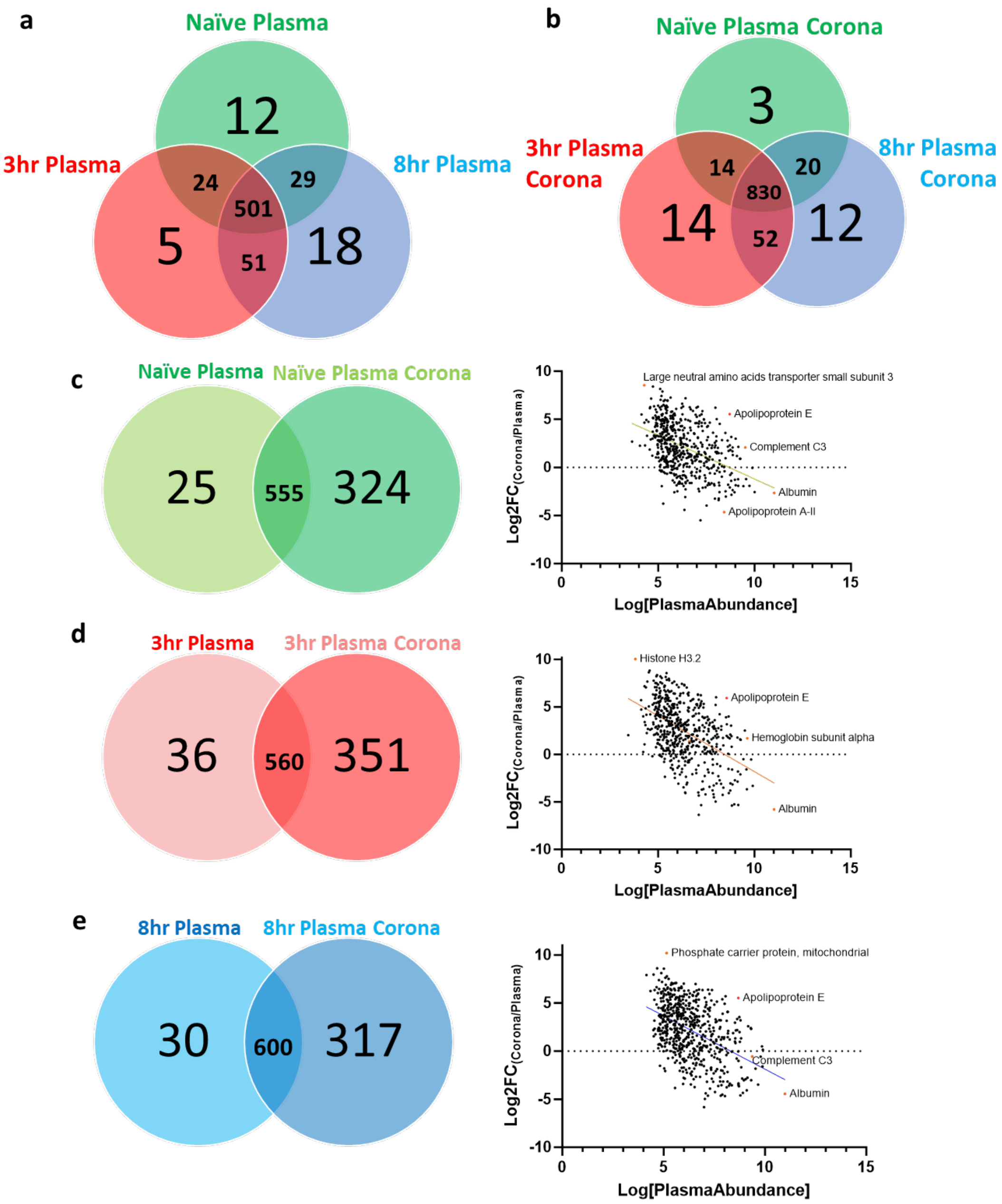


**Supplementary Fig. 6: NP coronas enrich low abundant plasma proteins**. Venn Diagram displaying the number of identified proteins in the plasma **(a)** and the NP corona **(b)** through proteomic analysis. **c-e.** Comparison of proteins identified in plasma versus proteins identified in NP coronas. Source data are provided as a Source Data file.

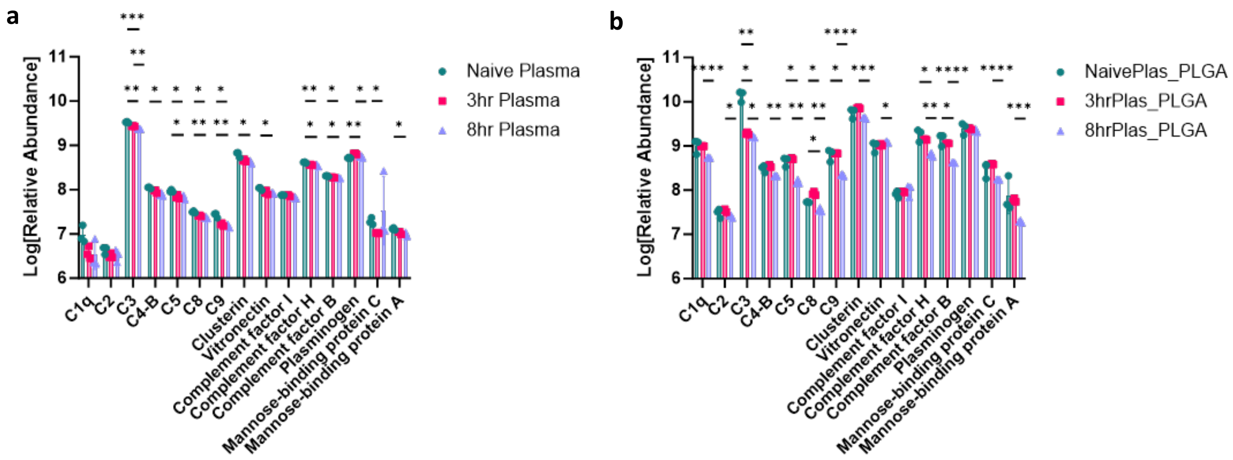


**Supplementary Fig. 7: Reduced complement adsorption in 8hrPlas NP coronas**. Individual complement factor relative abundances in whole plasma **(a)** or NP coronas **(b)**, obtained from mass spectrometry proteomic analysis. Significance was calculated using a Two-way ANOVA with Tukey's post-hoc test. *P < 0.05; **P < 0.01; ***P < 0.001, ****P < 0.0001. Comparisons without indicated bars are not significant. Source data are provided as a Source Data file.

| Accession # | Protein Name | kDa | NaïvePlas_PLGA | 3hrPlas_PLGA | 8hrPlas_PLGA |
|---|---|---|---|---|---|
| P08226 | Apolipoprotein E | 35.8 | 23990640211 ± 8536170831 | 22340996978 ± 750456128 | 22619594739 ± 573110145 |
| P02088 | Hemoglobin subunit beta-1 | 15.8 | 8283950457 ± 851143557 | 18459262370 ± 889772545 | 10017061711 ± 892437367 |
| P01942 | Hemoglobin subunit alpha | 15.1 | 6452125486 ± 445935904 | 13905777154 ± 755525098 | 8704875730 ± 467465705 |
| Q06890 | Clusterin | 51.6 | 5701410229 ± 1372485820 | 7258393830 ± 235075427 | 4296127875 ± 170976285 |
| O70362 | Phosphatidylinositol-glycan-specific phospholipase D | 93.2 | 6915380548 ± 2357237909 | 6646162332 ± 39350594 | 5895010713 ± 374190778 |
| Q8K0E8 | Fibrinogen beta chain | 54.7 | 3330932198 ± 151323838 | 5602500354 ± 118221621 | 10427623686 ± 432329813 |
| E9PV24 | Fibrinogen alpha chain | 87.4 | 3438392153 ± 104786140 | 5450391630 ± 218220451 | 10685664789 ± 120938590 |
| A6X935 | Inter alpha-trypsin inhibitor, heavy chain 4 | 104.6 | 2783598358 ± 596337668 | 5055553914 ± 68025198 | 1925277524 ± 89112465 |
| P11276 | Fibronectin | 272.4 | 3038841781 ± 553314030 | 5094975764 ± 203826148 | 5788516616 ± 344246366 |
| Q00623 | Apolipoprotein A-I | 30.6 | 7446085710 ± 478161107 | 4713063883 ± 377248000 | 4110944540 ± 121360700 |
| Q8VCM7 | Fibrinogen gamma chain | 49.4 | 2793887766 ± 220812174 | 4510125982 ± 246307221 | 8870528427 ± 233523722 |
| P97290 | Plasma protease C1 inhibitor | 55.5 | 3577771879 ± 1143304756 | 3615851557 ± 22808716 | 3100642531 ± 141751072 |
| Q01339 | Beta-2-glycoprotein 1 | 38.6 | 3707879327 ± 1281635550 | 3344533474 ± 291461274 | 2555576731 ± 257577820 |
| Q61646 | Haptoglobin | 38.7 | 1221472091 ± 177962141 | 2960846275 ± 294644036 | 1625095766 ± 150956894 |
| P20918 | Plasminogen | 90.7 | 2581269484 ± 745214142 | 2462843193 ± 89550547 | 2198497488 ± 188767060 |
| P01872 | Immunoglobulin heavy constant mu | 49.9 | 1819578950 ± 437250009 | 2385751147 ± 61027374 | 1658453571 ± 67558281 |
| P01027 | Complement C3 | 186.4 | 13994281401 ± 3692572652 | 1913515178 ± 124957988 | 1592100866 ± 70332155 |
| P07724 | Albumin | 68.6 | 16747339666 ± 22912449673 | 1906787138 ± 435393279 | 4473887836 ± 1039413798 |
| P13020 | Gelsolin | 85.9 | 1974607594 ± 352798467 | 1694590699 ± 53113167 | 464227732 ± 16142464 |
| P32261 | Antithrombin-III | 52 | 1490531372 ± 343759026 | 1490908840 ± 9986539 | 867095774 ± 56034628 |

**Supplementary Table 2: Top 20 proteins identified in PLGA NP Coronas.**

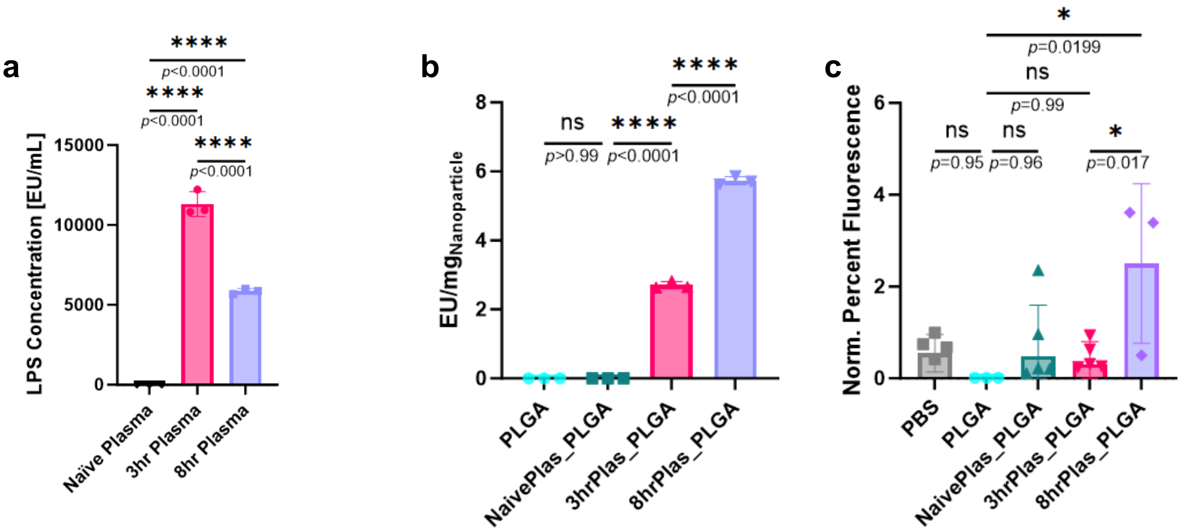


**Supplementary Fig. 8:** Quantification of LPS in whole plasma and NP coronas. **a.** Endotoxin quantification in whole plasma at varying timepoints post-LPS injection i.p (n = 3 mice). **b.** Endotoxin quantification in PLGA NP-coronas represented as EU/mg nanoparticle (n = 3 biological replicates). **c.** FITC-LPS fluorescence quantification on PLGA NPs when incubated in varying solutions (n = 6 biological replicates, except for PLGA and 8hrPlas_PLGA where n = 3). Significance was determined using a one-way ANOVA with Tukey's post-hoc test. Data sets are presented as mean ± S.D. *P < 0.05; **P < 0.01; ***P < 0.001, ****P < 0.0001. ns, not significant (P > 0.05). Source data are provided as a Source Data file.

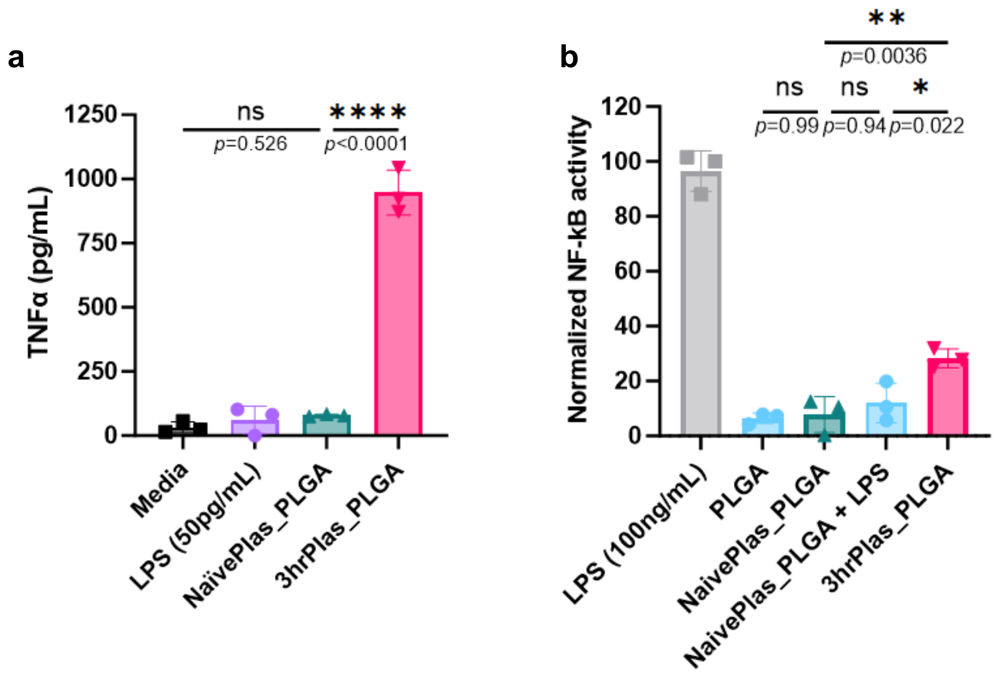


**Supplementary Fig. 9:** Evaluation of LPS contribution in NP corona-induced inflammation. **a.** BMDMs stimulated with LPS or NP coronas. **b.** Normalized NF-kB activity of RAW-Blue cells incubated with NP coronas. NaïvePlas_PLGA + LPS was created by supplementing Naïve plasma with 1µg/mL LPS prior to NP corona coating. Significance was determined using a one-way ANOVA with Tukey's post-hoc test. Data sets are presented as mean ± S.D of n = 3 biological replicates. *P < 0.05; **P < 0.01; ***P < 0.001, ****P < 0.0001. ns, not significant (P > 0.05). Source data are provided as a Source Data file.
