# Supplementary material for: Inflammatory disease progression shapes nanoparticle biomolecular corona-mediated immune activation profiles
Source: Nat Commun. 2025 Jan 22;16:924. doi: 10.1038/s41467-025-56210-4 (PMC11754911; doi:10.1038/s41467-025-56210-4)
Supplement: Supplementary file 2 — Reporting Summary [file 41467_2025_56210_MOESM2_ESM.pdf]

nature portfolio

Corresponding author(s): Ryan M. Pearson

Last updated by author(s): Dec 17, 2024

Reporting Summary

Nature Portfolio wishes to improve the reproducibility of the work that we publish. This form provides structure for consistency and transparency in reporting. For further information on Nature Portfolio policies, see our Editorial Policies and the Editorial Policy Checklist.

Statistics

For all statistical analyses, confirm that the following items are present in the figure legend, table legend, main text, or Methods section.

n/a	Confirmed	
[ ]	[x]	The exact sample size (*n*) for each experimental group/condition, given as a discrete number and unit of measurement
[ ]	[x]	A statement on whether measurements were taken from distinct samples or whether the same sample was measured repeatedly
[ ]	[x]	The statistical test(s) used AND whether they are one- or two-sided<br>*Only common tests should be described solely by name; describe more complex techniques in the Methods section.*
[x]	[ ]	A description of all covariates tested
[x]	[ ]	A description of any assumptions or corrections, such as tests of normality and adjustment for multiple comparisons
[ ]	[x]	A full description of the statistical parameters including central tendency (e.g. means) or other basic estimates (e.g. regression coefficient) AND variation (e.g. standard deviation) or associated estimates of uncertainty (e.g. confidence intervals)
[ ]	[x]	For null hypothesis testing, the test statistic (e.g. *F*, *t*, *r*) with confidence intervals, effect sizes, degrees of freedom and *P* value noted<br>*Give P values as exact values whenever suitable.*
[x]	[ ]	For Bayesian analysis, information on the choice of priors and Markov chain Monte Carlo settings
[x]	[ ]	For hierarchical and complex designs, identification of the appropriate level for tests and full reporting of outcomes
[x]	[ ]	Estimates of effect sizes (e.g. Cohen's *d*, Pearson's *r*), indicating how they were calculated

Our web collection on statistics for biologists contains articles on many of the points above.

Software and code

Policy information about availability of computer code

Data collection	Data collection was conducted using the instruments specified in the Methods.
Data analysis	Commercially available software used for data analysis are mentioned in the Methods: GraphPad Prism 10, FCS Express 7, Qiagen Ingenuity Pathway Analysis 24.0.1, MetaboAnalyst 5.0, MetIDQ, Agilent MassHunter Profinder v10, Proteome Discoverer v2.5.0.400, ImageJ v1.54h, Spectroflow v3.3.0

For manuscripts utilizing custom algorithms or software that are central to the research but not yet described in published literature, software must be made available to editors and reviewers. We strongly encourage code deposition in a community repository (e.g. GitHub). See the Nature Portfolio guidelines for submitting code & software for further information.

Data

Policy information about availability of data

All manuscripts must include a data availability statement. This statement should provide the following information, where applicable:

- Accession codes, unique identifiers, or web links for publicly available datasets
- A description of any restrictions on data availability
- For clinical datasets or third party data, please ensure that the statement adheres to our policy

The proteomics data generated in this study have been deposited in the PRIDE database under accession code PXD050918 [https://www.ebi.ac.uk/pride/archive/projects/PXD050918]. The lipidomics data generated in this study have been deposited in the Mendeley database [DOI: http://doi.org/10.17632/2z97z3bcw6.1]. The metabolomics data generated in this study have been deposited in the Mendeley database [DOI: http://doi.org/10.17632/4mfr9btjtt.1]. Source data is available

for Figures 1b, 1c, 1f, 1g, 2a, 2b, 2c, 2e, 2f, 3a, 3b, 3c, 3g, 3h, 3i, 4a, 4b, 4c, 4d, 5c, and Supplementary Figures 1b, 2a, 2b, 2e, 5, 7a, 7b, 8a, 8b, 8c, 9a, 9b in the associated source data file. Source data are provided as a Source Data file.

Research involving human participants, their data, or biological material

Policy information about studies with human participants or human data. See also policy information about sex, gender (identity/presentation), and sexual orientation and race, ethnicity and racism.

Reporting on sex and gender	Not applicable.
Reporting on race, ethnicity, or other socially relevant groupings	Not applicable.
Population characteristics	Not applicable.
Recruitment	Not applicable.
Ethics oversight	Not applicable.

Note that full information on the approval of the study protocol must also be provided in the manuscript.

Field-specific reporting

Please select the one below that is the best fit for your research. If you are not sure, read the appropriate sections before making your selection.

- [x] Life sciences
- [ ] Behavioural & social sciences
- [ ] Ecological, evolutionary & environmental sciences

For a reference copy of the document with all sections, see nature.com/documents/nr-reporting-summary-flat.pdf

Life sciences study design

All studies must disclose on these points even when the disclosure is negative.

Sample size	Samples sizes were estimated based on similar reports in literature and prior research from our lab. All sample sizes used are indicated in the manuscript. Endotoxemia mouse studies were conducted as n=10 mice per group and plasmas were pooled to ensure homogeneity representative of the inflammation state in subsequent corona analyses. Flow cytometry samples were measured as triplicates of >8000 events. For in vitro cell experiments, data represents a minimum of n=3 biological replicates.
Data exclusions	No data was excluded.
Replication	To ensure reproducibility, experiments were independently performed at least thrice. In addition to different batches of synthesized NPs to verify synthesis parameters do not effect the biological responses observed. All attempts at replication were successful.
Randomization	Animals were randomly assigned to groups to minimize bias and ensure proper representation across groups.
Blinding	No blinding of data was applied because the readouts were objective.

Reporting for specific materials, systems and methods

We require information from authors about some types of materials, experimental systems and methods used in many studies. Here, indicate whether each material, system or method listed is relevant to your study. If you are not sure if a list item applies to your research, read the appropriate section before selecting a response.

Materials & experimental systems

n/a	Involved in the study	
[ ]	[x]	Antibodies
[ ]	[x]	Eukaryotic cell lines
[x]	[ ]	Palaeontology and archaeology
[ ]	[x]	Animals and other organisms
[x]	[ ]	Clinical data
[x]	[ ]	Dual use research of concern
[x]	[ ]	Plants

Methods

n/a	Involved in the study	
[x]	[ ]	ChIP-seq
[ ]	[x]	Flow cytometry
[x]	[ ]	MRI-based neuroimaging

Antibodies

Antibodies used	All antibodies were purchased from BioLegend (San Diego, CA): anti-CD16/32 (1:500, Cat No. 101302, Clone 93), anti-mouse CD11b-Pacific Blue (1:500, Cat No. 101224, Clone M1/70), F4/80-PE/Cyanine7 (1:500, Cat No. 123114, Clone BM8), CD86-APC/Cyanine7 (1:666.66, Cat No. 105030, Clone GL-1), CD80-FITC (1:100, Cat No. 104706, Clone 16-10A1), PD-L1-PE (1:200, Cat No. 124308, Clone 10F.9G2)
Validation	All primary antibodies were purchased from the supplier as noted above and used without additional validation. The validation of all the antibodies could be found from manufacturer BioLegend online.

Eukaryotic cell lines

Policy information about cell lines and Sex and Gender in Research

Cell line source(s)	RAW-Blue™ cells were purchased from InvivoGen (San Diego, CA).
Authentication	Cell lines were used as received without further validation.
Mycoplasma contamination	Cell lines were not tested for mycoplasma.
Commonly misidentified lines (See ICLAC register)	No commonly misidentified cell lines were used.

Animals and other research organisms

Policy information about studies involving animals; ARRIVE guidelines recommended for reporting animal research, and Sex and Gender in Research

Laboratory animals	Male and female C57BL/6 mice (5-7 weeks old) were purchased from the University of Maryland Veternary Resources. TLR4-/- K/O C57BL/6J (B6(Cg)-Tlr4tm1.2Karp/J) mice (5-7 weeks) were gifted by S. Vogel Lab at University of Maryland, Baltimore (Baltimore, MD). All animals were maintained on a standard diet and water.
Wild animals	The study did not involve wild animals.
Reporting on sex	The majority of this study was completed in female mice, however results were recapitulated in male mice comparators.
Field-collected samples	The study did not involve field-collected samples.
Ethics oversight	All protocols were approved by the Institutional Animal Care and Use Committee at the University of Maryland, Baltimore under protocol AUP-00000975.

Note that full information on the approval of the study protocol must also be provided in the manuscript.

Plants

Seed stocks	Not applicable.
Novel plant genotypes	Not applicable.
Authentication	Not applicable.

Flow Cytometry

Plots

Confirm that:

- [x] The axis labels state the marker and fluorochrome used (e.g. CD4-FITC).
- [x] The axis scales are clearly visible. Include numbers along axes only for bottom left plot of group (a 'group' is an analysis of identical markers).
- [x] All plots are contour plots with outliers or pseudocolor plots.
- [x] A numerical value for number of cells or percentage (with statistics) is provided.

Methodology

Sample preparation	Treated macrophage cells were collected and washed using PBS containing 1% FBS through centrifugation (600g x 5min) at 4C. Cell were then Fc-blocked with anti-CD16/32 antibody (1:100 dilution)for 10 minutes at 4C. Following blocking, cells were stained with an antibody master-mix containing anti-CD-11b, F4/80, CD86, CD80, PDL-1 antibodies for 15 minutes in the dark at 4C. Afterwards,antibodies were washed off with FBS supplemented PBS and Live/Dead stained with propidium iodide before flow cytometry analysis.
Instrument	Cytek Aurora flow cytometer
Software	Spectroflow software was used to collect the data and FCS Express was used to analyze.
Cell population abundance	A minimum of 8000 cells were analyzed for each sample and samples were taken in biological triplicates.
Gating strategy	Gating strategy is shown in the supplemental information. Briefly, a gate was drawn around all cells. Single cells are determined with the forward scatter (FSC) height and area. Cells were then identified as macrophages through CD11b and F4/80 positivity. PI negativity was used to determine live cell gating, then live cells were quantified for the different co-receptor markers CD80, CD86, and PD-L1.

☒ Tick this box to confirm that a figure exemplifying the gating strategy is provided in the Supplementary Information.
